# Foraging Behavior of Subantarctic Fur Seals Supports Efficiency of a Marine Reserve’s Design

**DOI:** 10.1371/journal.pone.0152370

**Published:** 2016-05-10

**Authors:** Stephen P. Kirkman, Dawit G. Yemane, Tarron Lamont, Michael A. Meÿer, Pierre A. Pistorius

**Affiliations:** 1 Department of Environmental Affairs, Branch Oceans and Coasts, Cape Town, South Africa; 2 Fisheries Management, Department of Agriculture, Forestry and Fisheries, Cape Town, South Africa; 3 Department of Zoology, Nelson Mandela Metropolitan University, Port Elizabeth, South Africa; New York Institute of Technology College of Osteopathic Medicine, UNITED STATES

## Abstract

Foraging behaviour of marine top predators is increasingly being used to identify areas of ecological importance. This is largely enabled by the ability of many such species to forage extensively in search of prey that is often concentrated in oceanographically productive areas. To identify important habitat in the Southern Indian Ocean within and around South Africa’s Prince Edward Islands’ Marine Protected Area (MPA), satellite transmitters were deployed on 12 lactating Subantarctic fur seals *Arctocephalus tropicalis* at Prince Edward Island (PEI) itself. Switching state space models were employed to correct ARGOS tracks and estimate behavioural states for locations along predicted tracks, namely travelling or area restricted search (ARS). A random forest model showed that distance from the study colony, longitude and distance from the Subantarctic Front were the most important predictors of suitable foraging habitat (inferred from ARS). Model-predicted suitable habitat occurred within the MPA in relatively close access to the colony during summer and autumn, but shifted northwards concurrently with frontal movements in winter and spring. The association of ARS with the MPA during summer-autumn was highly significant, highlighting the effectiveness of the recently declared reserve’s design for capturing suitable foraging habitat for this and probably other marine top predator species.

## Introduction

Quantifying habitat use of animals is vitally important for understanding their biophysical requirements, such as nutrition and reproduction, but also for predicting areas of ecological significance [1–3]. Breeding and foraging grounds are especially important for conservation as those areas constitute crucial habitats in animals’ lifecycles [4]. In this regard, numerous studies have documented relationships between the foraging areas of marine predators, mainly surface-feeding seabirds and epipelagic foraging marine mammals, and specific oceanographic processes and features that influence their prey distribution [5]. In particular, foraging areas have been characterized by sea-surface temperature [6, 7], surface chlorophyll-a concentration [8,9], frontal features [10,11] and bathymetric features [10, 12]. Such information on the distribution and habitat use is highly useful for conservation and management efforts such as mitigating human impact and adequately delimiting marine protected areas (MPAs) and for providing insights into the potential impact of climate change [13, 14].

The Prince Edward Islands Marine Protected Area (PEI MPA) which was recently proclaimed in South Africa’s Southern Ocean territorial waters was created in part to sustain the foraging requirements of the large seabird and seal populations breeding on the islands of the Prince Edward Islands Archipelago (PEIA), including Prince Edward Island itself, and Marion Island [15]. Foraging areas of southern elephant seals *Mirounga leonina* and of wandering *Diomedea exulans* and grey-headed albatrosses *Thalassarche chrysostoma* were taken into account in the design of the reserve, which consists of three axes extending from the islands in the centre to the boundary of the exclusive economic zone (EEZ). The average position of oceanic fronts which were shown to be important foraging areas was also considered. MPA boundaries have been advocated to be dynamic in the long-term in response to climate-mediated oceanographic changes such as possible southwards shifts in the positions of the important oceanic fronts in the Southern Ocean [16] and improved understanding of the associated marine ecosystem.

A recommendation following from the PEI MPA design process [15] was that foraging patterns of seabirds and seals should continue to be monitored to enable detection of biotic responses to climate change that could have relevance to the appropriateness of reserve boundaries. In this regard a lack of information on foraging areas of fur seals *Arctocephalus* spp. was identified as an important knowledge gap. Subsequently, foraging areas have been documented for fur seals from Marion Island [10], but to date there has been no published research on foraging distribution of any of the marine top predator species breeding on Prince Edward Island. This is of concern when considering that conspecific populations breeding within the same archipelago often have very different foraging distributions (e.g. [17, 18]).

To improve our understanding of foraging requirements of marine top predators breeding at Prince Edward Island, and assess this in relation to the current PEI MPA, this study focuses on the foraging distribution of Subantarctic fur seal *A*. *tropicalis* females breeding at Prince Edward Island. Specifically, the aims of the study were to (a) Describe baseline foraging parameters of lactating *A*. *tropicalis* from Prince Edward Island; (b) Characterise their foraging areas by determining the association of foraging areas with relevant physical and environmental oceanographic habitat features, including sea surface temperature (SST), chlorophyll-*a* (Chl-*a*) concentrations, sea surface height anomalies (SSHA) and locations of frontal and bathymetric features; (c) Predict suitable foraging areas for *A*. *tropicalis* from Prince Edward Island in space and time (season) based on the distribution of oceanographic habitat; and (d) Compare predictions with the current boundaries of the PEI MPA.

## Materials and Methods

### Study site

**Fig 1:**
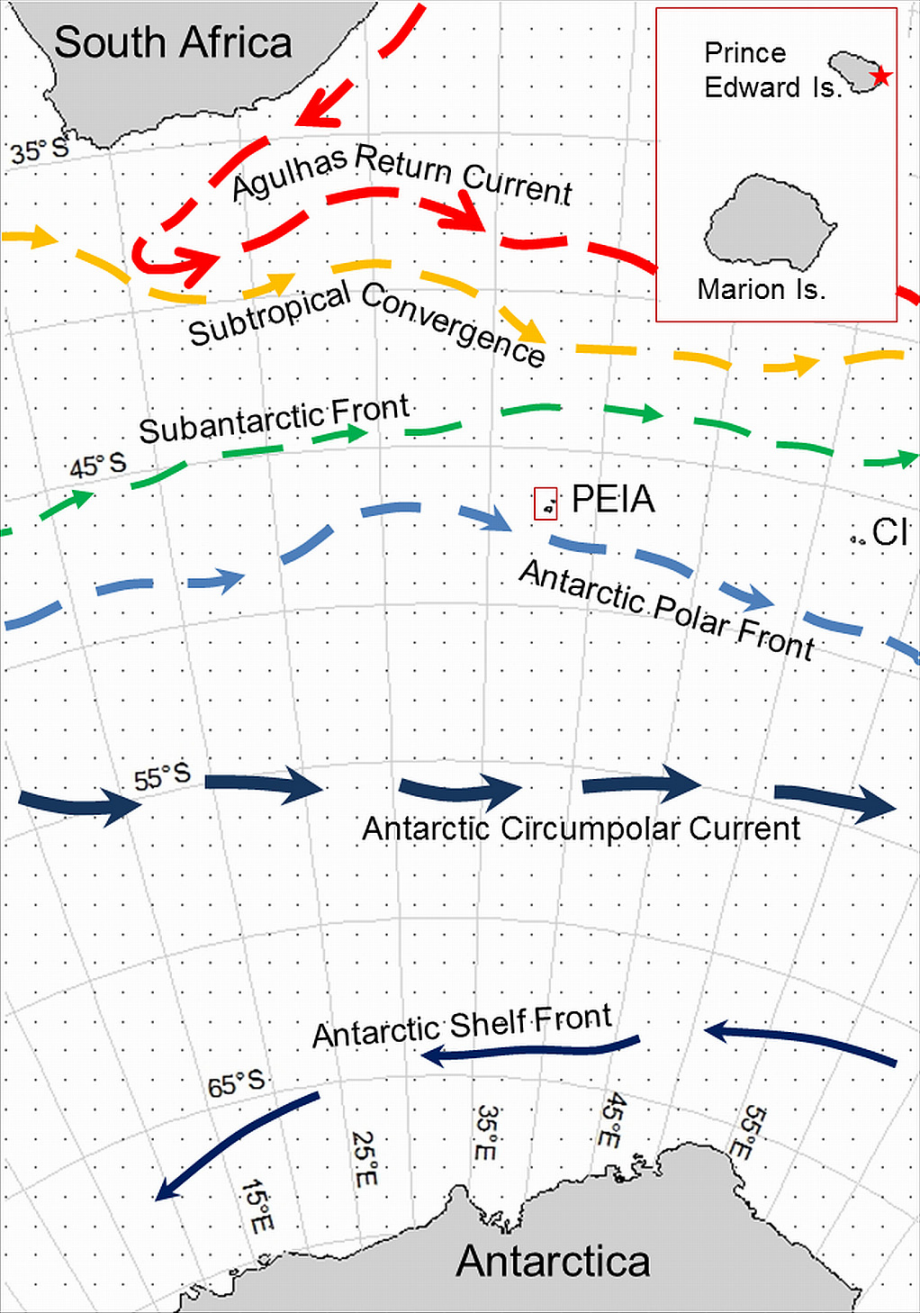
The location of the Prince Edward Islands in relation to fronts and currents in the Southern Ocean region between Africa and Antarctic. The location of the study area, Cave Bay, marked in the insert.

Field work took place on PEI (46°38’S, 37°57’E), which is situated about 1 400 km south of South Africa and 19 km to the north of Marion Island (Fig 1), during March 2011. The two islands are located in the highly dynamic oceanic environment between the Subantarctic Front (SAF) and the Antarctic Polar Front (APF). Breeding sites of *A*. *tropicalis* are largely concentrated on the east coast of PEI, with only a few suitable beaches to be found on the exposed and cliff-dominated west coast [19]. Cave Bay, where the deployments were made, is a large indentation in the south-eastern coastline of PEI (Fig 1).

### Field work

Twelve lactating female *A*. *tropicalis* observed to be suckling a pup were captured with a modified hoop net [20]. The females were selected randomly from the grassy slopes behind the boulder beaches, where capturing was relatively easy. After opening a dorsal aperture in the cone of the net while the seal was physically restrained, a satellite platform terminal transmitter (PTT) was attached to the fur on the dorsal midline of the seal immediately posterior to the scapulae, using a double-component, quick-setting epoxy resin (Araldite AW2101, CIBA-GEIGY Ltd). The PTTs were Spot 5 tags (Wildlife Computers, Redmond, WA) that relayed location data to the ARGOS Satellite System.

Fresh scat samples were collected from the study site and stored individually for subsequent sorting and identification of hard part remains. In the laboratory the scat samples were washed through a 0.5-mm sieve under running water to collect the undigested prey remains. Fish otoliths (sagittae) and cephalopod beaks (lower beaks) were used to identify prey remains to the lowest possible taxonomic level by an experienced person using identification guides [21–23]. The frequency of occurrence of each taxonomic group and its percentage contribution to the diet by numerical abundance were determined.

All fieldwork was permitted by the Department of Environmental Affair’s Branch Oceans and Coasts, the management authority of South Africa’s marine and coastal environment including the Prince Edward Islands, with ethics approval previously granted for seal capture and immobilisation in South Africa and its subantarctic territories by the former Marine and Coastal Management Animal Ethics Committee. The ARGOS tracking data are available at South Africa’s Department of Environmental Affairs’ Oceans and Coastal Research Marine Information Management System repository (www.data.ocean.gov.za).

### Data analysis

#### State space models

State space models (SSMs) are viewed as among the most sophisticated new tools for analyzing animal movement and migration from electronic tracking data [24]. ARGOS satellite tags impose complex error structures on tracking data [25] that must be dealt with appropriately so that important biological variability can be separated from artificial noise [24]. Filtering of data by use of e.g. speed filters or simply omitting poor quality location classes (e.g. B and Z) are subjective and discard substantial amounts of potentially valuable data, but also do not correct the location error in the remaining data (e.g. [26, 27]). Fitting of SSMs, which separately account for ARGOS location class error structure and stochasticity in animal movement using correlated random walk models (CRWs), present a more parsimonious approach to dealing with observation error in animal tracking data [28,29]. Irregular, non-Gaussian error distributions are incorporated into this complex statistical framework using Markov Chain Monte Carlo (MCMC) estimation methods, and the most likely locations are predicted without removing extreme observations. In switching SSMs (SSSMs), two or more sets of parameters, each representing a discrete behavioural state, are estimated for a CRW, by utilising movement properties such as turn angles, move lengths and autocorrelation [28]. Different distributions are generated for each set of parameters and the probability of the animal being in one of the alternative states is provided for every location in the predicted track.

We corrected the ARGOS tracks and estimated behavioural states for each location along the predicted tracks using the “DCRWS” (First Difference Correlated Random Walk with Switching) state space model [30] available in the bsam (Bayesian State Space Models for Animal Movement) 0.45 package in the R statistical environment [31]. Only two behavioural states were specified (to avoid too much model complexity), which we termed “travelling” and “area restricted search” (ARS). The latter implies slowing down of movement and remaining for longer in areas, theoretically areas of higher prey density. To take into account the temporal resolution of the ARGOS data and at the same time avoiding excessive computational times, we decided on a time-step of four hours between locations when performing the analysis. The MCMC estimation for each dataset (i.e. locations for each individual seal) had a burn-in of 10000 samples followed by 50000 actual samples that were thinned by a factor of 10 resulting in an effective sample size of 5000. Attributes of foraging trips, including duration and length (km) of trip, the furthest distance reached from the study colony, and the percentage of time spent in ARS, were calculated from the predicted tracks and behavioural states of the SSSMs.

#### Bathymetric and hydrographic data

Using ETOPO1, bathymetric data were extracted for a grid of sufficient size to include the furthest tracks from the study colony, i.e. between 9°E, 34S° in the Northwest to 56°E, 51.5°S in the Southeast. Seasonal (three-monthly) averages over the entire grid were generated for remotely-sensed SST and Chl-*a* using MODIS Aqua 9km resolution, and for SSH anomalies (SSHA) from the new merged AVISO product with a 20-year reference period [32, 33]. Commencing with the month of deployment, seasons were categorised as follows: Autumn = March-May (MAM), Winter = June-August (JJA), Spring = September-November (SON), Summer = December-February (DJF). Interpolation of missing SST and Chl-*a* data was performed using block kriging.

Previously mean locations of the Subtropical Convergence (STC), Subantarctic Front (SAF), and Antarctic Polar Front (APF) were described as 41.6±1.07°S, 46.4±1.07°S, and 50.3±1.33°S, respectively, using hydrographic data [34]. In this study, similar to Durgadoo et al. [35], the surface locations of the STC, SAF and APF were identified by the seasonal average locations of the 14°C, 8°C, and 4°C isotherms, respectively.

#### Analysing habitat preference as a function of environmental variables

Two approaches were used to relate ARS of seals to sets of environmental variables and predict the most suitable habitat for foraging, namely Random Forests (RF) [36] and Generalised Boosted Regression Models (GBM) [37]. Both are ensemble learning methods in which final predictions are made by aggregating predictions from a number of individual models (decision trees). GBM is an example of the “boosted” approach to ensemble learning, which is a sequential approach whereby each subsequent tree seeks to minimize residuals weighted by the previous tree's errors (a shrinkage parameter) and in the end a weighted vote determines the prediction. RF is a type of “bagging” approach whereby successive trees do not depend on earlier trees, instead each is independently constructed using a bootstrapped sample of the data set, and in the end a simple majority vote is taken for the prediction. RF add an additional layer of randomness to bagging by using a modified tree learning algorithm that selects, at each candidate split in the learning process, a random subset of the features. Thus if one or a few features are very strong predictors for the response variable, these features will be selected in many of the trees, causing them to become correlated.

GBM was applied using the GBM package [38] and RF using the randomForests package [39] in R. In each case the response variable was the DCRWS predicted behavioural state (i.e. ARS and travelling) and each predicted location. The predictor variables included in the initial models were longitude (lon), latitude (lat) depth, season, the extracted SST and SSHA values, distance from the colony (distance), distance from the STC (distance14; taken as the nearest straight line distance in km between the predicted location and the isoline of the STC in the same season) and distance from the SAF (distance8; taken as the nearest straight line distance in km between the predicted location and the average location of the SAF in the same season); distance from the APF was not considered because there was very little movement to the south of the islands. Chl-*a* was not included in the models because in autumn and winter the spatial data gaps resulting from extensive and persistent cloud cover, were too great for reliable interpolation of missing values south of 40°S. Also, especially given location errors associated with ARGOS positions, it was not always possible to distinguish between ARS in waters close to the island and residency at the study colony. Consequently it was decided to exclude all locations within 50 km of the study colony from the GBMs and RF, to avoid confounding of ARS with residency. GBMs and RF were used to predict suitable foraging habitat over the entire grid, based on the relationships in the above models. Other packages used for the visualisation and analysis of the data included reshape2 [40], ggplot2 [41], Plyr [42] and ggmap [43]. The predictive performance of the models was assessed using re-sampling, specifically the “Leave Group out Cross Validation' LGOCV” approach (also known as Monte-Carlo cross validation) whereby each model is repeatedly trained on a subset of data, in this case 80% of the data, to evaluate the remaining subset [44]. The area under the receiver operating characteristic (ROC) curve (AUC) was used as the performance measure [45]. ROC is a general purpose method that was designed, based on the values of a continuous variable, to determine a threshold above which it is indicated that an event occurred. In the context of classification, regarding two classes as in this case (ARS vs non-ARS activity), ROC is the plot of

Sensitivity/(1−Specificity)

Sensitivity in the context of this study is the proportion of correctly classified ARS locations and specificity is the proportion of correctly classified non-ARS locations, therefore the denominator is the proportion of false ARS (locations incorrectly classified as ARS while they are in fact non-ARS). AUC for a classification model measures how well the model correctly classifies/discriminates between the classes considered (here ARS and non-ARS activities). A model with higher AUC is generally considered to be the best classifier compared to a random classifier (which has AUC of 0.5).

Calculation of the relative importance of predictors for the RF was based upon changes in predictive error. The prediction error was calculated for each tree based on the data that were not used in the training of the model (i.e. the “out-of bag sample”), following which the predictors were permuted and a prediction error rate was calculated. The difference between the prediction error was averaged across all trees and normalized by the standard deviation of the difference to provide the relative importance of each predictor. Considering only the area within the EEZ of the islands (200 nm), a Pearson’s chi-square test was performed on the 2x4 contingency table of the frequency of ARS within vs outside the MPA, to test whether or not the distribution of ARS in relation to the MPA boundaries was associated with season. Then chi-square tests with Bonferroni corrections were conducted for each season and were weighted by the size difference between the area inside and the area outside the MPA (inside = 180 288 km^2^ excluding the areas of the islands, outside = 347 386 km^2^; [15]). These tests were done to assess whether or not the frequency of ARS depends on the size of the area inside vs outside the MPA.

## Results

Overall, 87 completed foraging trips were recorded from the twelve study females (Table 1). One female (ID number 57349) behaved aberrantly soon after deployment, relocating to Marion Island, and her data were disregarded for the summary statistics of foraging trips. From the remaining 11 females, 85 trips were measured (Table 1), including 57 trips during autumn, 14 in winter, 7 in spring and 9 in summer (where trips extended between seasons they were allocated to the season within which the longest period of the trip occurred). The instruments of three of these eleven animals only provided data for autumn, three provided data only up to winter, one only up to spring, three provided a full seasonal cycle of data from autumn to summer, while one (ID 66384) left on an extended trip at the beginning of winter and did not return until the breeding season, when her transmitter failed. The mean duration of trips per season ranged from 13 days (summer) to 95 days (spring). The mean distance travelled from the colony and the mean round trip distance, per season, extended from 167 km (summer) to 998 km (spring), and from 391 km (summer) to 4 401 km (spring) (Table 1).

**Table 1:** Statistics (mean, standard deviation, minimum and maximum) summarising attributes of completed foraging trips (duration, distance travelled from study site and round trip distance) by individual study animals, per season at Prince Edward Island (2011–2012). Where trips extended between seasons they were allocated to the season within which the longest period of the trip occurred. An X in the ID field indicates a female which behaved aberrantly, relocating to Marion Island shortly after deployment. This female is omitted from the “All combined” statistics at the end of the table.

ID	Season	No. of trips	Duration (days)				Furthest distance (km) from PEI				Round trip distance (km)			
			Mean	S.d.	Min	Max	Mean	S.d.	Min	Max	Mean	S.d.	Min	Max
56101	Autumn	2	20	2	18	22	496	182	367	625	1298	578	890	1707
57349^X^	Autumn	2	49	25	31	67	872	421	574	1170	2984	1243	2105	3863
66297	Autumn	3	19	3	16	22	501	65	435	564	1307	147	1141	1421
66306	Autumn	4	16	4	13	22	294	73	200	372	814	174	570	975
	Winter	1	20		20	20	260		260	260	1048		1048	1048
66307	Autumn	7	11	3	7	16	202	63	110	266	531	156	263	743
	Winter	2	14	3	12	16	320	49	285	355	838	185	707	969
	Spring	1	174		174	174	1540		1540	1540	9087		9087	9087
	Summer	2	13	3	11	16	272	20	258	286	630	29	609	650
66308	Autumn	5	14	2	11	17	339	99	212	446	841	182	624	1024
	Winter	1	15		15	15	317		317	317	806		806	806
66314	Autumn	5	13	3	8	16	252	85	103	318	686	250	272	888
	Winter	1	14		14	14	290		290	290	865		865	865
66346	Autumn	4	18	5	12	25	487	170	378	738	1142	369	861	1680
	Winter	2	54	18	41	67	1238	32	1215	1260	3336	709	2835	3838
	Spring	1	85		85	85	824		824	824	3375		3375	3375
66348	Autumn	6	12	3	7	15	295	137	118	497	795	408	328	1459
66357	Autumn	5	12	3	9	17	269	118	155	406	706	255	446	1021
	Winter	4	20	7	14	31	374	127	207	487	1024	325	651	1309
	Spring	2	48	47	15	82	485	127	395	575	2405	1873	1081	3729
	Summer	5	282	168	119	566	6	3	4	12	68	2	66	70
66383	Autumn	5	12	2	9	16	276	78	150	356	666	186	360	867
	Winter	3	31	20	15	54	251	12	240	263	1224	684	635	1975
	Spring	2	52	24	35	69	551	198	411	691	1969	997	1264	2674
	Summer	2	7	4	5	10	188	95	121	255	423	236	256	590
66384	Autumn	9	6	3	2	11	120	90	22	330	315	210	49	792
	Spring	1	204		204	204	2552		2552	2552	9598		9598	9598
All	Autumn	57	13	4	2	25	283	137	22	738	730	339	49	1707
combined	Winter	14	25	17	12	67	445	345	207	1260	1346	932	635	3838
	Spring	7	95	70	15	204	998	787	395	2552	4401	3519	1081	9598
	Summer	9	8	4	4	16	167	87	55	286	391	207	119	650

**Fig 2:**
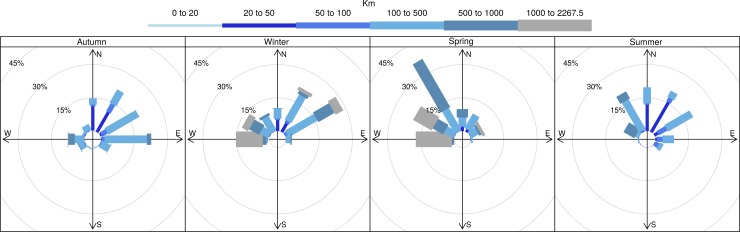
Distance rose plots summarising the distances, within different direction classes, of all positions of the adult Subantarctic fur seal females tagged at Prince Edward Island (n = 12), from the study colony during the study period (March 2011-Feb 2012).

**Fig 3:**
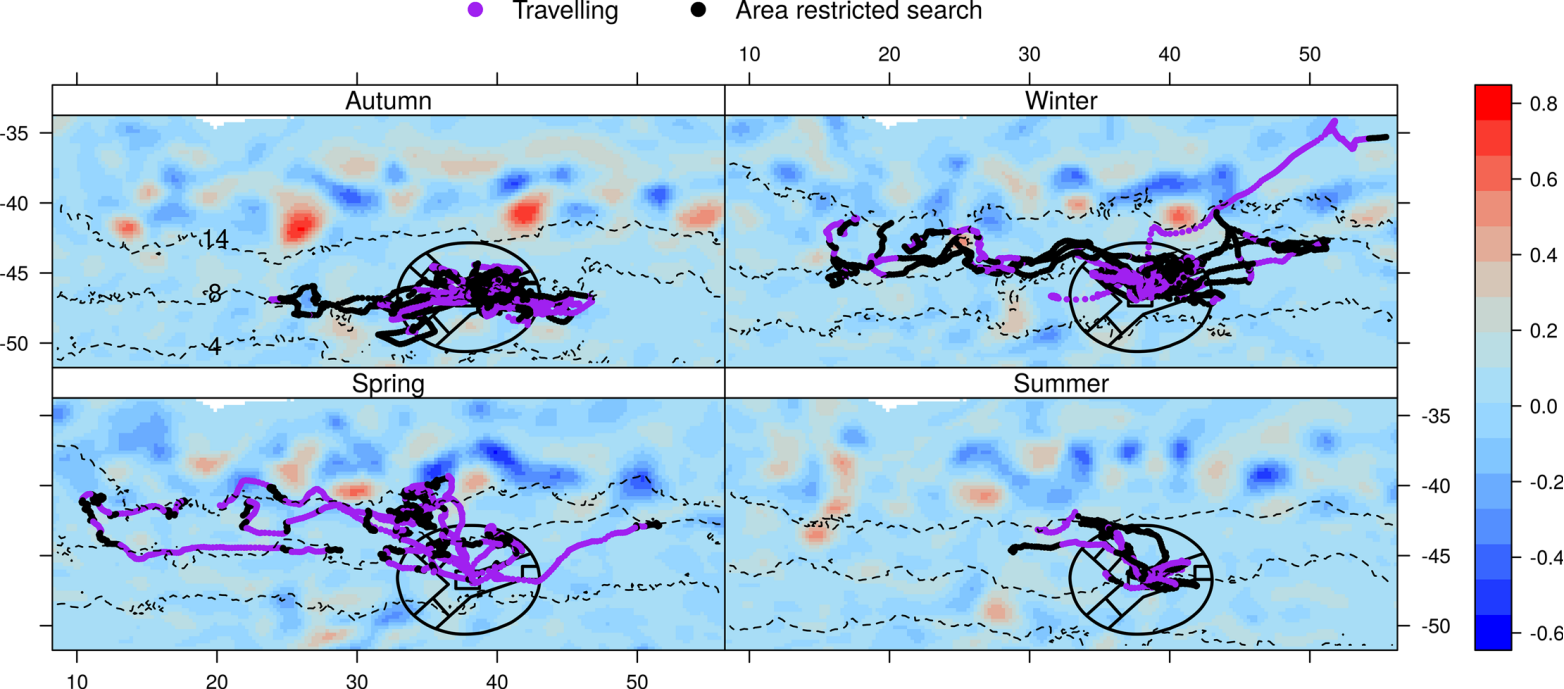
**Switching state space model predicted tracks of adult Subantarctic fur seal females tagged at Prince Edward Island in March 2011, overlaid on seasonal averages of sea surface height anomaly for (A) Autumn (March-May; n = 12 seals), (B) Winter (June-August; n = 8 seals), (C) Spring (September-November; n = 6 seals), (D) Summer (December-February; n = 4 seals).** The segments of predicted tracks that were associated with area restricted search (ARS) behaviour are distinguished from those associated with travelling. The dashed lines show the average surface locations of the Subtropical Convergence (STC), Subantarctic Front (SAF), and Antarctic Polar Front (APF), identified by the 14°C, 8°C, and 4°C sea surface temperature isotherms, respectively.

**Fig 4:**
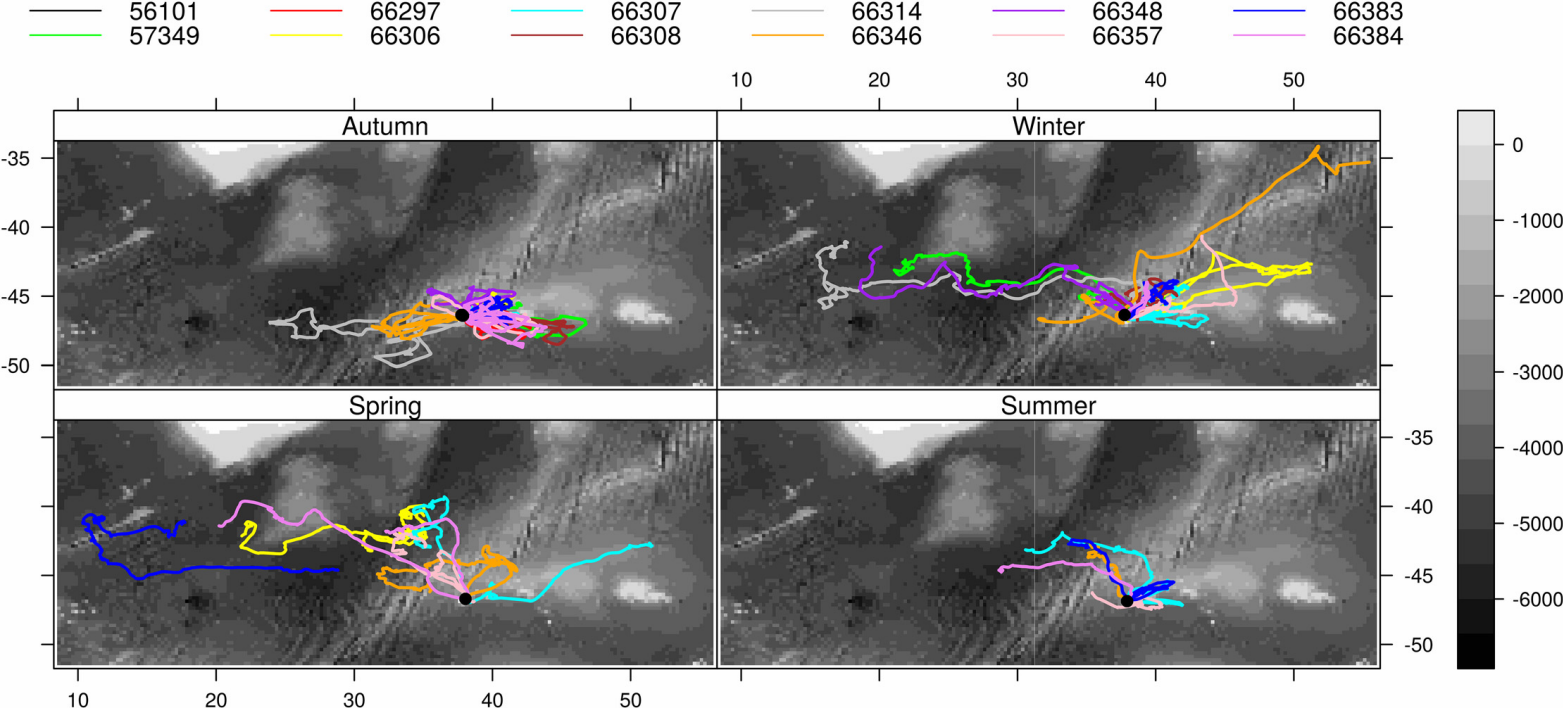
Switching state space model predicted tracks of the adult Subantarctic fur seal females (n = 12) tagged at Prince Edward Island in March 2011 and tracked between then and February 2012, overlaid on bathymetry (m). The individual seals identified by their PTT numbers are in the legend; the black circle is the study site.

**Fig 5:**
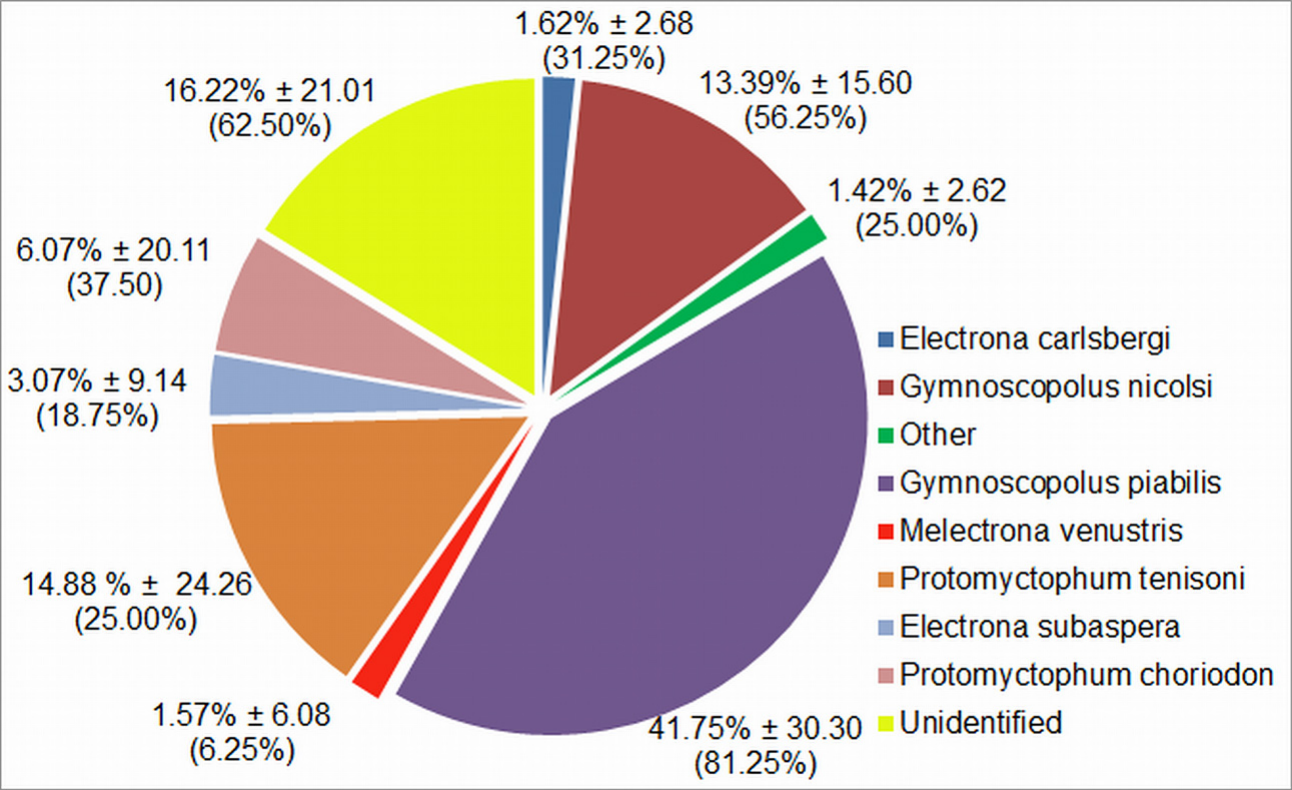
The mean relative abundances (± standard deviations) of fish prey items per scat collected from Prince Edward Island in March 2011 (n = 16). The percentage frequency of occurrence of each prey item is given in parentheses.

The most ARS occurred to the east and northeast of the island, followed by the west and northwest both coinciding with arms of the MPA (Figs 2 and 3 and S1 and S2). Summer and autumn were associated with shorter trip durations and distances than winter and spring and were also the periods when the mean surface location of the SAF was in closest proximity to the islands (Table 1, Figs 2 and 3), i.e. 259 km and 277 km, respectively, compared with 427 km and 416 km in winter and spring. In winter and spring, when the mean surface locations of the SAF were further to the north, the trips were generally also further to the north (Figs 2 and 3) and were associated with greater trip durations and distances (Table 1). ARS during extended trips of spring were frequently in the vicinity of the STC (Fig 3). Some ARS still occurred in this vicinity during early summer, before remaining tagged females returned to breed (e.g. Fig 4D). No foraging occurred to the south of the islands approximately between bearings of 120° and 240° of the islands (Figs 2 and 3) and no ARS was associated with the surface location of the APF. During summer and autumn, ARS corresponded with shallower areas (rises or ridges) especially to the east of the islands (Fig 4). The prey information retrieved from a small sample of scats (n = 16) which were collected at the time of deployment during autumn showed that myctophid fish species dominated the prey contents (Fig 5). *Gymnoscopolus piabilis* followed by *Protomyctophum tenisoni* and *P nicholsi* were the most abundant species found.

**Fig 6:**
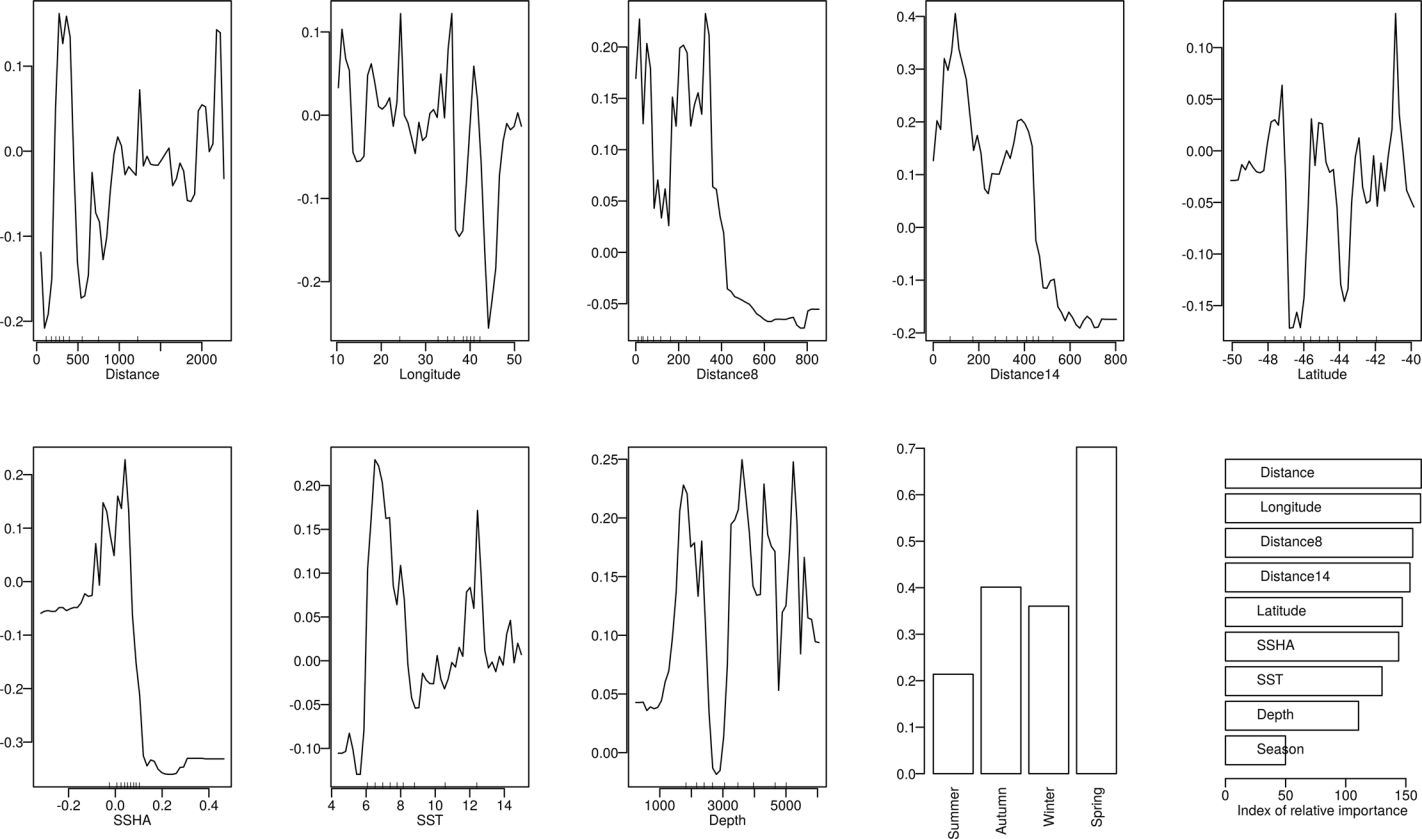
Random forest model response curves for the different predictors of behavioural activity of the twelve adult Subantarctic fur seal females tagged at Prince Edward Island in March 2011 and tracked between then and February 2012. Distance represents distance from the study colony, Distance8 is the distance from the Subantarctic Front and Distance14 is the distance from the Subtropical Convergence Zone. The final panel shows the relative importance of all the predictors in terms of their influence on the predictive accuracy of the model.

**Fig 7:**
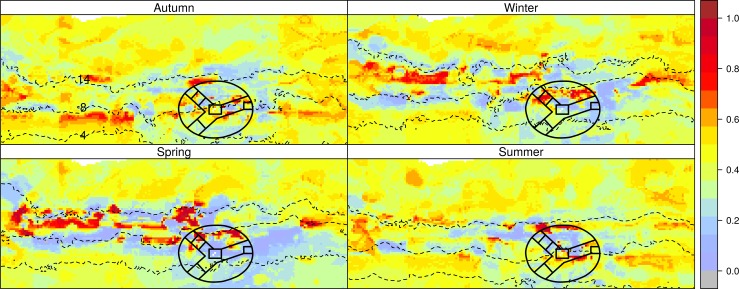
Foraging habitat suitability (preferred areas for ARS) in the study domain for Subantarctic fur seals *Arctocephalus tropicalis* in each of the four seasons as predicted by a random forest model with behavioural state (travelling or area restricted search) as a function of geographical and environmental variables. The dashed lines show the average surface locations of the Subtropical Convergence (STC), Subantarctic Front (SAF), and Antarctic Polar Front (APF), identified by the 14°C, 8°C, and 4°C sea surface temperature isotherms, respectively.

In terms of the AUC, the predictive performance of the RF model was greater than that of the GBM model (Mean predictive AUC for RF = 0.98, for GBM = 0.95; S3 and S4 Figs). Therefore only the RF model results are presented in the main body, but corresponding GBM results are presented in S5 and S6 Figs). The response curves for most of the predictors of the behavioural activity of seals were largely non-linear (Fig 6). The preferred areas for ARS varied over the ranges of longitude and latitude suggesting patchy distribution of ARS. The depth at preferred areas was intermediate at about 1500 m or at greater depths over 3500 m; shallower depths were not preferred. They tended to prefer areas that were further from the island, closer to the SAF (distance8) than the STC (distance14). This is also clearly illustrated in Fig 3. Areas preferred for ARS were characterised by SST in the range of 6–8°C and 11–13°C, which is close to the average temperatures of the above-mentioned fronts. There were two peaks associated with the response to SSHA (Fig 6), one positive (around 0.05) and the other negative (around -0.05). While these numbers are small because of the seasonal averaging, this suggests the association of the tracks with both cyclonic and anticyclonic eddies, which are formed and travel eastward along the fronts. Distance from the colony, distance from the fronts, longitude and SSHA were the most important predictors of ARS (Fig 6). The model predictions of habitat suitability (Fig 7), performed for each season separately, show the northwards shift of the predicted suitable areas (preferred areas for ARS) during winter and spring when they showed less spatial overlap with the MPA compared with autumn, and retracting further southward again during summer.

During autumn, the season when deployments took place, 55% of total ARS behaviour occurred within the MPA boundaries according the SSSM results, and a further 31% outside of the MPA within the EEZ of the PEIs (i.e. with 200 nm radius of the islands). The corresponding percentages were 18% and 33% for winter, 9% and 28% for spring, and 34% and 37% in summer. Considering only the area within the EEZ of the islands, the Pearson’s chi-square test of the 2x4 contingency table of the frequency of ARS within vs outside the MPA by season showed that the distribution of ARS in relation to the MPA boundaries was significantly associated with season (*χ*^2^ = 279.6, *df* = 3, *P* < 0.001). Posthoc chi-square tests (with Bonferroni correction for multiple testing) for each season showed that during autumn and summer, ARS was significantly more likely to occur within the MPA than outside it and the opposite was true for winter while the result for spring was not significant (Table 2).

**Table 2:** Results of chi-square tests with Bonferroni corrections to test whether or not the frequency of ARS depends on the size of the area inside vs outside the MPA, in each season. In the test, the frequencies were weighted by the size differences between the MPA and non-MPA areas (0.34: 0.66).

Season	ARS inside MPA	ARS outside MPA	*χ*^2^	*P*
Autumn	1069	574	706.52	< 0.001
Winter	485	734	18.19	< 0.001
Spring	194	412	1.06	1.00
Summer	195	166	64.46	< 0.001

## Discussion

We here presented results on the first year-round tracking study on *A*. *tropicalis* and the first from South Africa’s Prince Edward Island. The directionality and distance of foraging trips varied by season and appeared to be influenced by the migrating fronts. SSHA, possibly associated with eddies around the fronts was an important predictor of ARS. Although these targeted features are largely ephemeral in nature, there was a clear seasonal overlap between ARS and the PEI MPA during the course of the study indicating its importance in protecting suitable foraging habitat for *A*. *tropicalis* and potentially also of other marine top predator species breeding on Prince Edward Island.

The ARGOS tracks collected in this study were corrected using state space modelling—it has been shown that tracks corrected using SSMs provide substantial improvements compared with tracks based on raw or filtered ARGOS locations [4]. One of two discrete behavioural states, namely travelling or ARS, was then predicted for each location based on movement properties such as turn angles, move lengths and autocorrelation, using switching state space modelling. ARS implies slowing down of movement and remaining for longer in areas with inferred higher prey density. Based on a study of the benthic foraging Australian fur seal *A*. *pusillus doriferus*, Hoskins et al. [46] caution against inferring foraging activity from 2D tracking data alone because fine-scale foraging activity can be confused with resting behaviour at the surface.” It is uncertain whether this applies to pelagic foraging fur seal species such as *A*. *tropicalis*. Notwithstanding this, the Hoskins et al. [46] study was a fine-scale analysis of the distribution of foraging effort, whereas the present study based on ARGOS data is at a coarser scale, but given that resting behaviour would generally be associated with foraging activity, major misrepresentations in the predicted locations of foraging are considered unlikely. However, inclusion of the underwater aspect of at-sea movements is recommended for further investigations of this population

De Bruyn et al. [10] first reported on movements of lactating *A*. *tropicalis* adults from Marion Island that were tracked during periods in summer and also in autumn/winter. Study animals tended to forage eastwards of the island, while other top predators from the island including *M*. *leonina* had been shown to move predominantly westwards (e.g. [47, 48]). The authors were surprised at the long distances of foraging trips (> 400 km), considering that *A*. *tropicalis* from two other locations at a similar latitude or further south, namely Îles Crozet and Macquarie Island respectively, fed within close proximity (< 100 km) of the study sites [49–51]. As reported by de Bruyn et al. [10] and subsequently by Wege [52] for Marion Island *A*. *tropicalis*, foraging activity of PEI *A*. *tropicalis* occurred mainly to the east and north east of the islands, with less animals foraging to the west and north west and very little foraging activity to the south of the islands (Fig 2). Interestingly, this is despite the model predictions of suitable habitat availability to the northwest and west of the islands (Fig 7). The species composition and abundance of myctophid fish in the contents of the scats of the Prince Edward Island seals, collected at the time of deployment (Autumn), also matched results of previous studies of *A*. *tropicalis* at Marion Island [10, 53]. This indicates similar prey composition between the neighbouring islands, although the diet results of this study should be treated with caution because of the small sample of scats collected (n = 16). Of the three most prominent species found in the diet, one (*G*. *piabilis*) is generally distributed to the south of about 46°S and the other two (*P*. *tenisoni* and *P*. *nicholsi*) between the STC and the APF [22]. Foraging behaviour in terms of the duration of trips (see Table 1) was also similar to the Marion Island population, where significantly longer trips were reported for winter than summer or autumn [54, 55].

Although *A*. *tropicalis* rarely dive deeper than 200 m [56], Wege [52] showed that Marion Island *A*. *tropicalis* females dived every night following departure from the island and therefore assumed that they foraged opportunistically *en route* to preferred foraging areas. According to de Bruyn et al. [10], increased opportunity for such feeding during travelling in the shallower waters to the east of the island (~2500 mean depth) compared with the west (~4500 mean depth) could account for the preference of the former. This is because the shallower area to the east of the island which is characterised by elevated bathymetric features results in flow dynamics of differing bodies of water that are conducive for a shallow mixed layer with conditions more suitable to productivity near the surface than over the deeper benthos to the west [57, 58]. More specifically, substantial mixing of warmer SAF waters and colder APF waters that takes place to the east of the islands (downstream), together with nutrient output from the islands creates an area conducive to phytoplankton growth and zooplankton assemblages from both cooler and warmer waters [58–60]. While areas with bottom depths of ~1500 were preferred for ARS by the animals in this study, areas of greater depths (> 3500 m) were also favoured (Fig 6): shallower areas were preferred during summer and autumn but seasonal variation in ARS which generally shifted further northwards during winter and spring indicates that sufficient prey availability in the shallower areas is not persistent throughout the year, as illustrated in the model predictions of habitat suitability (Fig 7). The location of the fronts seems to be an important determinant of ARS, with the northward shift in ARS corresponding with a northwards shift of the fronts, with most activity associated with the SAF but as from spring also with the STC (Figs 3 and 6). Interestingly winter and spring are also the periods when *A*. *tropicalis* individuals have mostly been recorded as vagrants on the shores of the African continent or associated islands ([61–64], MAM unpublished data).

Depth was much less important than the relative positions of the fronts in terms of the predictive accuracy of the RF model (Fig 6). There is considerably higher mesoscale variability related to frontal systems than in other regions of the Southern Ocean [35, 57, 65–69], and the distribution and abundance of marine top predators, especially seabirds, have been shown to be affected strongly by fronts [70]. Whether this is because of the enhanced primary production of frontal systems or because they concentrate prey into exploitable patches, these features have been shown to be important determinants of prey capture in the open ocean where prey aggregations are typically unpredictable.

Spring was characterised by the greatest distance and duration of trips (Table 1). For lactating females the selected foraging area is a compromise between body condition and the energetic needs of the pup, whereas after weaning around September-October [71] they are not constrained by having to return to the breeding colony allowing more distant foraging. This is potentially important in order to maximise energy intake prior to giving birth again. During their incubation period (November-December), when they are also not constrained by the energetic needs of offspring, breeding *T*. *chrysostoma* tracked from Marion Island also tended to forage in the STC [48], which is often characterised by considerable levels of mesoscale variability associated with the formation of eddies and periodically enhanced levels of productivity. The Subtropical Convergence and warmer water further north of it were also frequented by non-breeding *D*. *exulans* from Marion Island [72] and the STC is a favoured foraging area of *A*. *tropicalis* from Amsterdam Island during their lactation period [73]. Like their conspecific from the Prince Edwards islands, these *A*. *tropicalis* undertake long-distance foraging migrations and lengthy at-sea time during lactation periods [56, 73].

The identification and characterisation of top predator foraging areas is increasingly being recognised for providing useful information for spatial-based marine conservation planning and fisheries management [74]. It has consequently been adopted in several studies aimed at delineating appropriate locations or boundaries for spatial protection or to assess the effectiveness of protected areas in the near shore or the open ocean (e.g. [75–78]. In the case of the Prince Edward Island MPA for example, the design of the reserve took into account the foraging distributions of *M*. *leonina*, *D*. *exulans* and *T*. *chrysostoma* that were tracked from Marion Island, as well as the average position of oceanic fronts which were shown to be important foraging areas. The design of the PEI MPA, which consists of three axes extending from the islands in the centre to the EEZ boundary, maximizes the chances of incorporating shifting positions of the oceanic fronts and therefore capture of important foraging areas of apex predators, by traversing latitudinal and longitudinal gradients [15]. Lack of information on foraging areas of fur seals [15] and of PEI marine top predators in general [79] have previously been identified as information gaps. While the *A*. *tropicalis* populations at the Prince Edward Islands have recovered from past overexploitation and the species is not currently of conservation concern [80], information on their at-sea distribution and behaviour is useful for verifying reserve boundary adequacy and setting baselines for further monitoring. Continued monitoring can enable detection of biotic responses to climate change that could have relevance to the effectiveness of reserve boundaries [15]. As per the one of the aims of this study, it was shown statistically that the proportion of ARS (assumed to represent foraging activity) that occurred within the reserve boundaries especially during autumn but also in summer was significantly greater than the proportion distributed in the remainder of the EEZ. These seasons corresponded with model predictions of increased habitat suitability in the vicinity of the islands (Fig 7), and foraging trips of relatively short distance and duration relative to the other seasons (Table 1). The accessibility of suitable foraging habitat for lactating females at these times of the year is vital because the fasting ability of the pups is limited [81], compared with winter when the pups are older and the females are able to undertake extended foraging trips to suitable habitat which at that time is predicted to be at greater distances from the islands (Fig 7). The results therefore support the effectiveness of the reserve design for capturing suitable foraging habitat for this species at a critical period of their annual cycle. More years of telemetry data would be useful for assessing heterogeneity in habitat suitability for this species between years and its effects on foraging area fidelity, and for modelling implications of predicted climate change impacts such as a southward shifts in the locations of fronts [16] on the adequacy of the reserve design.

## Supporting Information

S1 Fig **Switching state space model predicted tracks of adult Subantarctic fur seal *Arctocephalus tropicalis* females tagged at Prince Edward Island in March 2011, overlaid on seasonal averages of sea surface temperature (°C) for (A) Autumn (March-May; n = 12 seals), (B) Winter (June-August; n = 8 seals), (C) Spring (September-November; n = 6 seals), (D) Summer (December-February; n = 4 seals).** The segments of predicted tracks that were associated with area restricted search (ARS) behaviour are distinguished from those associated with travelling. The dashed lines show the average surface locations of the Subtropical Convergence (STC), Subantarctic Front (SAF), and Antarctic Polar Front (APF), identified by the 14°C, 8°C, and 4°C sea surface temperature isotherms, respectively.
(TIF)

S2 Fig **Switching state space model predicted tracks of adult Subantarctic fur seal *Arctocephalus tropicalis* females tagged at Prince Edward Island in March 2011, overlaid on seasonal averages of chlorophyll-*a* for (A) Autumn (March-May; n = 12 seals), (B) Winter (June-August; n = 8 seals), (C) Spring (September-November; n = 6 seals), (D) Summer (December-February; n = 4 seals).** The segments of predicted tracks that were associated with area restricted search (ARS) behaviour are distinguished from those associated with travelling. The dashed lines show the average surface locations of the Subtropical Convergence (STC), Subantarctic Front (SAF), and Antarctic Polar Front (APF), identified by the 14°C, 8°C, and 4°C sea surface temperature isotherms, respectively.
(TIF)

S3 Fig The performance of the random forest model in terms of correctly predicting suitable foraging habitat (assumed to be associated with area restricted search) for Subantarctic fur seals *Arctocephalus tropicalis* at Prince Edward Island, on the training set, based on the Area Under the Curve (AUC) of the Receiver Operating Characteristic (ROC) curve. Sensitivity is the proportion of correctly classified area restricted search (ARS) locations and specificity is the proportion of correctly classified non-ARS locations, therefore 1—specificity is the proportion of false ARS (locations incorrectly classified as ARS while they are in fact non-ARS).
(TIF)

S4 Fig The performance of the generalised boosted regression model in terms of correctly predicting suitable foraging habitat (assumed to be associated with area restricted search) for Subantarctic fur seals *Arctocephalus tropicalis* at Prince Edward Island, on the training set, based on the Area Under Curve (AUC) of the Receiver Operating Characteristic (ROC) curve. Sensitivity is the proportion of correctly classified area restricted search (ARS) locations and specificity is the proportion of correctly classified non-ARS locations, therefore 1—specificity is the proportion of false ARS (locations incorrectly classified as ARS while they are in fact non-ARS).
(TIF)

S5 Fig Generalised boosted regression model response curves for the different predictors of behavioural activity of the twelve adult Subantarctic fur seal *Arctocephalus tropicalis* females tagged at Prince Edward Island in March 2011 and tracked between then and February 2012. Distance represents distance from the study colony, Distance8 is the distance from the Subantarctic Front and Distance14 is the distance from the Subtropical Convergence Zone. The final panel shows the relative importance of all the predictors in terms of their influence on the predictive accuracy of the model.
(TIF)

S6 Fig Foraging habitat suitability (preferred areas for ARS) in the study domain for Subantarctic fur seals *Arctocephalus tropicalis* in each of the four seasons as predicted by a generalised boosted regression model with behavioural state (travelling or area restricted search) as a function of geographical and environmental variables. The dashed lines show the average surface locations of the Subtropical Convergence (STC), Subantarctic Front (SAF), and Antarctic Polar Front (APF), identified by the 14°C, 8°C, and 4°C sea surface temperature isotherms, respectively.
(TIF)
