# Supplementary figures and images for: Foraging Behavior of Subantarctic Fur Seals Supports Efficiency of a Marine Reserve’s Design
Source: PLoS One. 2016 May 10;11(5):e0152370. doi: 10.1371/journal.pone.0152370 (PMC4862747; doi:10.1371/journal.pone.0152370)

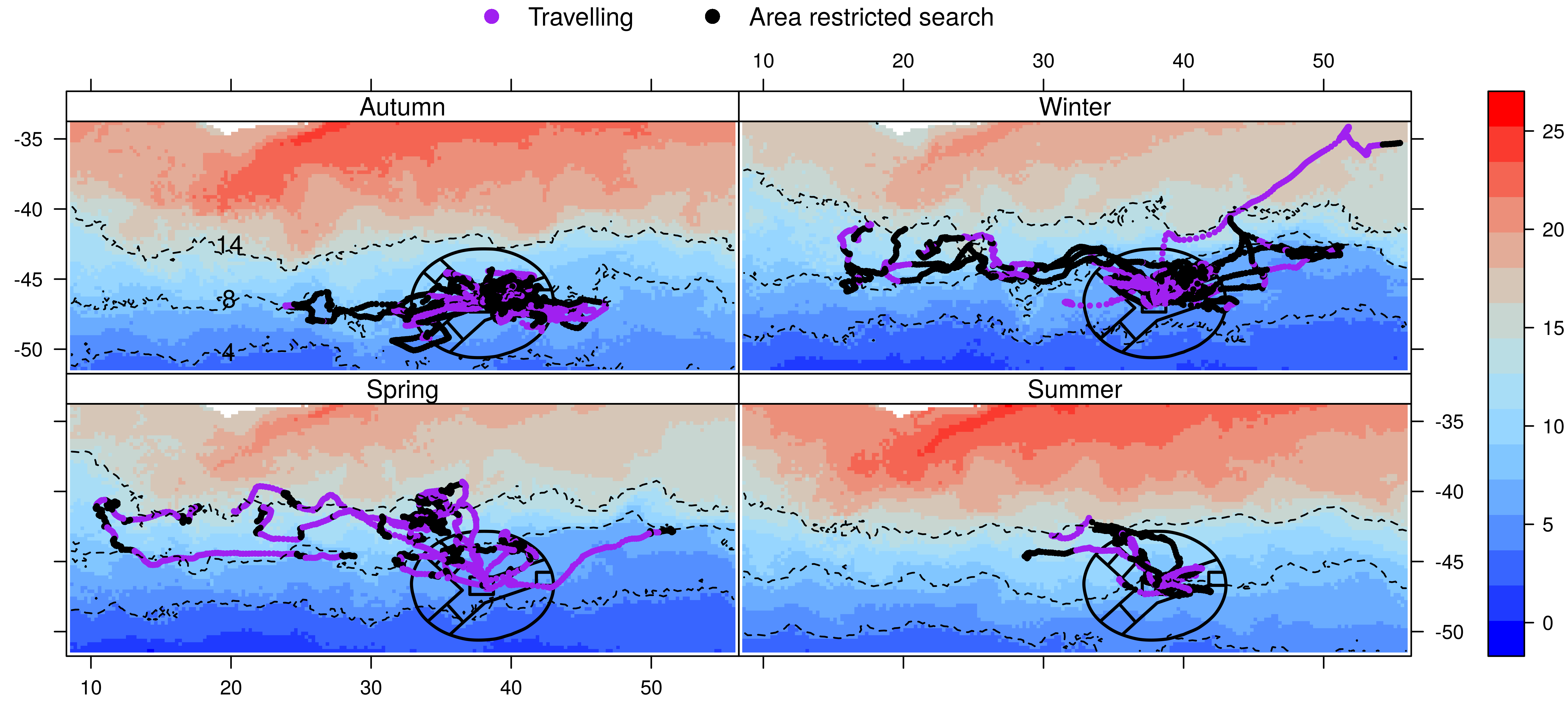

Supplement: S1 Fig — Switching state space model predicted tracks of adult Subantarctic fur seal Arctocephalus tropicalis females tagged at Prince Edward Island in March 2011, overlaid on seasonal averages of sea surface temperature (°C) for (A) Autumn (March-May; n = 12 seals), (B) Winter (June-August; n = 8 seals), (C) Spring (September-November; n = 6 seals), (D) Summer (December-February; n = 4 seals). The segments of predicted tracks that were associated with area restricted search (ARS) behaviour are distinguished from those associated with travelling. The dashed lines show the average surface locations of the Subtropical Convergence (STC), Subantarctic Front (SAF), and Antarctic Polar Front (APF), identified by the 14°C, 8°C, and 4°C sea surface temperature isotherms, respectively. (TIF) [file pone.0152370.s001.tif]

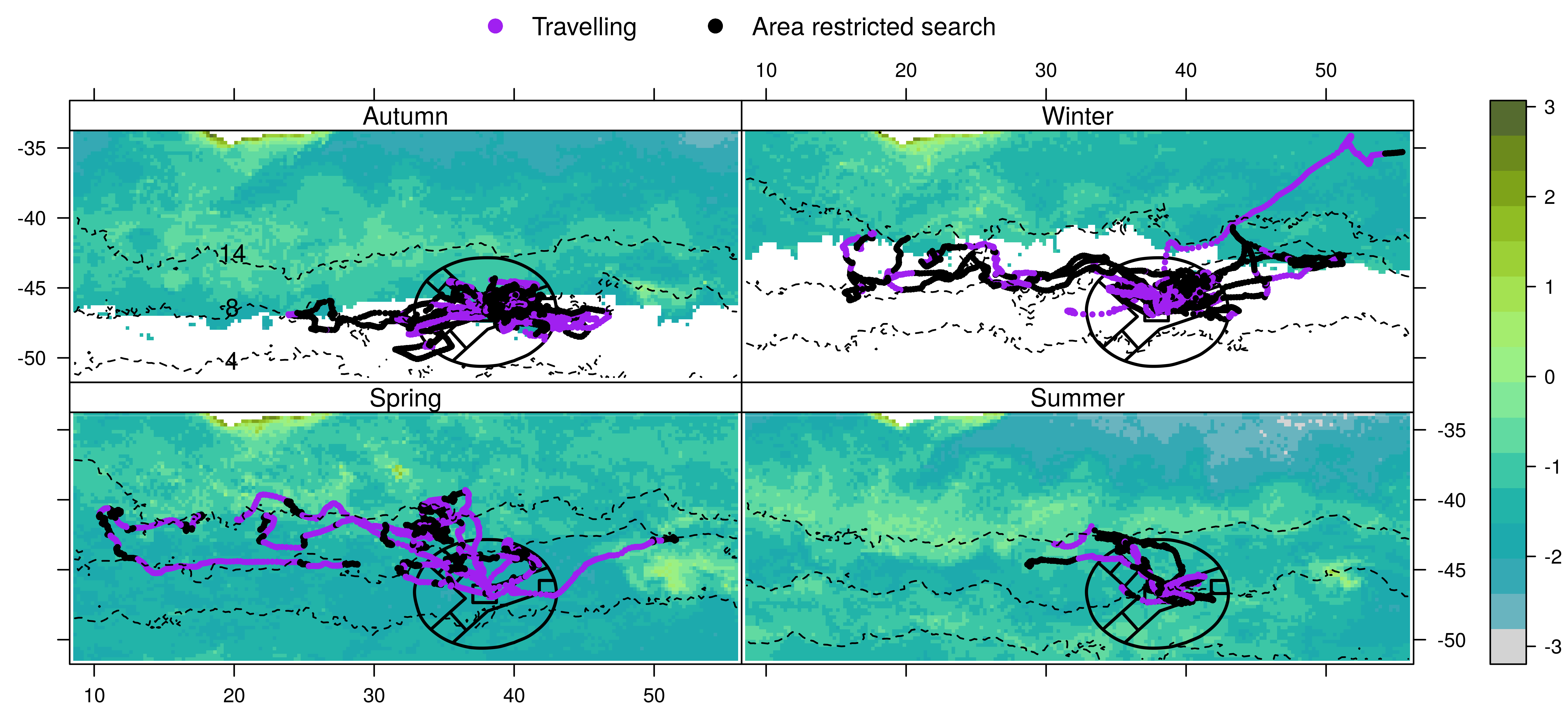

Supplement: S2 Fig — Switching state space model predicted tracks of adult Subantarctic fur seal Arctocephalus tropicalis females tagged at Prince Edward Island in March 2011, overlaid on seasonal averages of chlorophyll-a for (A) Autumn (March-May; n = 12 seals), (B) Winter (June-August; n = 8 seals), (C) Spring (September-November; n = 6 seals), (D) Summer (December-February; n = 4 seals). The segments of predicted tracks that were associated with area restricted search (ARS) behaviour are distinguished from those associated with travelling. The dashed lines show the average surface locations of the Subtropical Convergence (STC), Subantarctic Front (SAF), and Antarctic Polar Front (APF), identified by the 14°C, 8°C, and 4°C sea surface temperature isotherms, respectively. (TIF) [file pone.0152370.s002.tif]

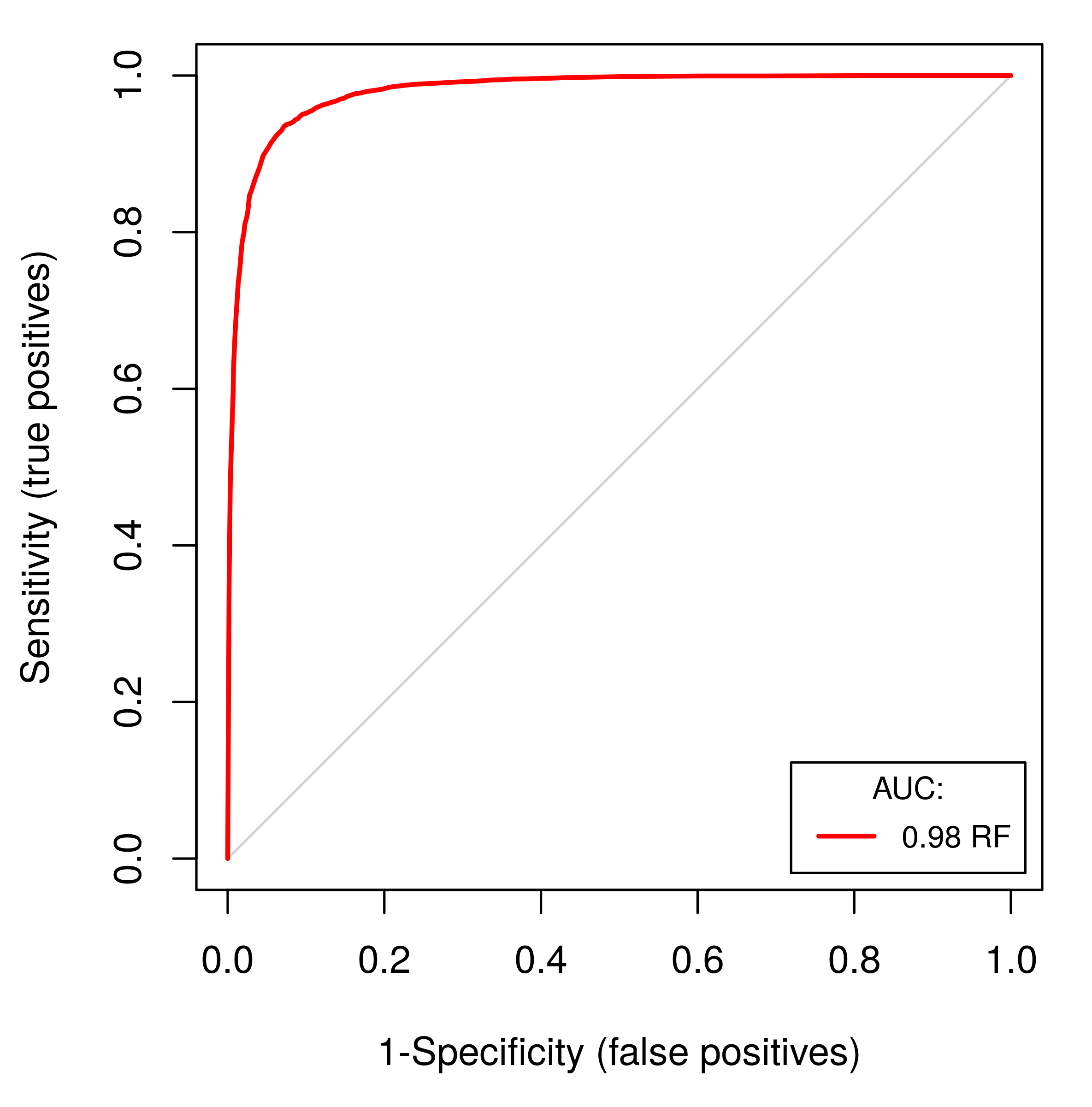

Supplement: S3 Fig — Sensitivity is the proportion of correctly classified area restricted search (ARS) locations and specificity is the proportion of correctly classified non-ARS locations, therefore 1—specificity is the proportion of false ARS (locations incorrectly classified as ARS while they are in fact non-ARS). (TIF) [file pone.0152370.s003.tif]

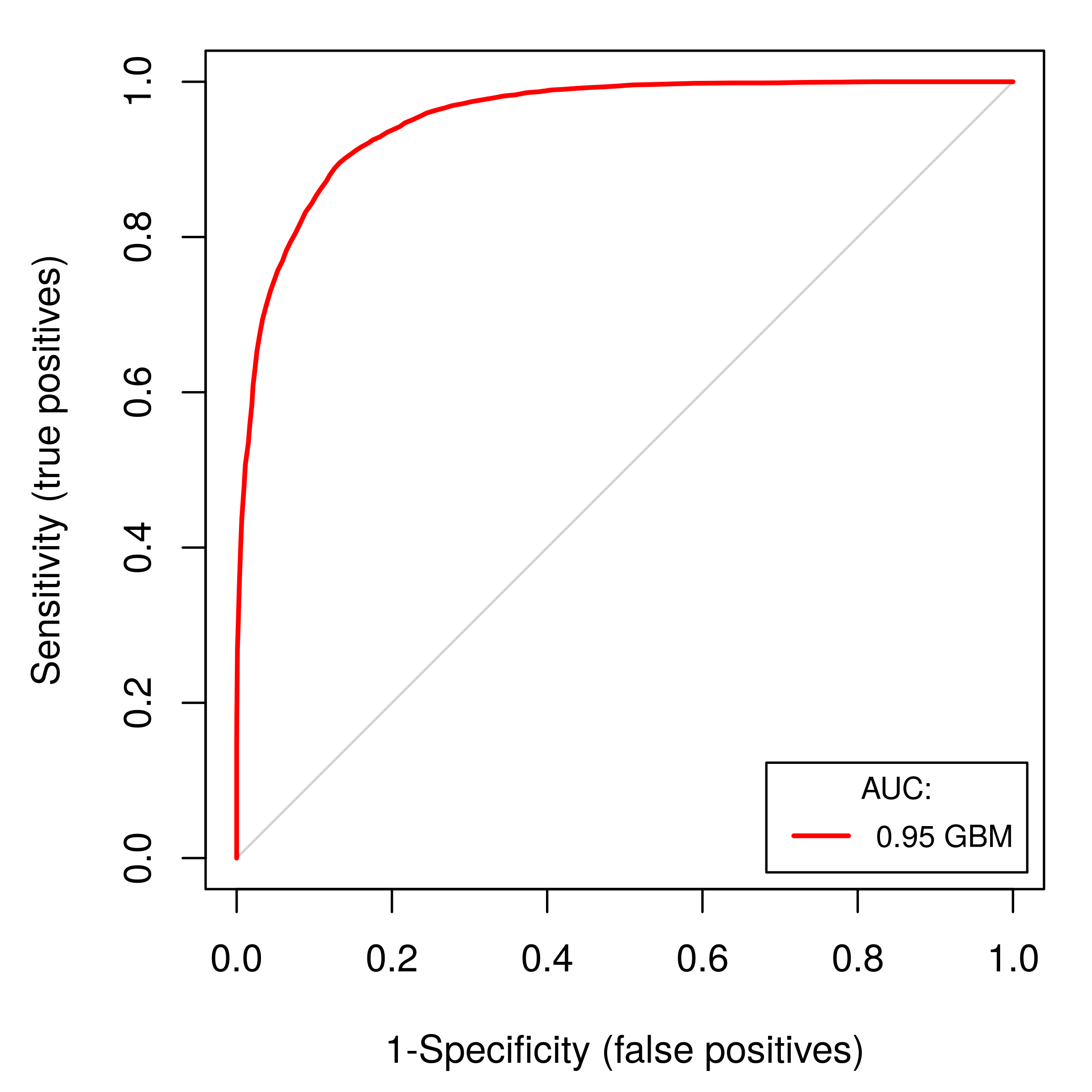

Supplement: S4 Fig — Sensitivity is the proportion of correctly classified area restricted search (ARS) locations and specificity is the proportion of correctly classified non-ARS locations, therefore 1—specificity is the proportion of false ARS (locations incorrectly classified as ARS while they are in fact non-ARS). (TIF) [file pone.0152370.s004.tif]

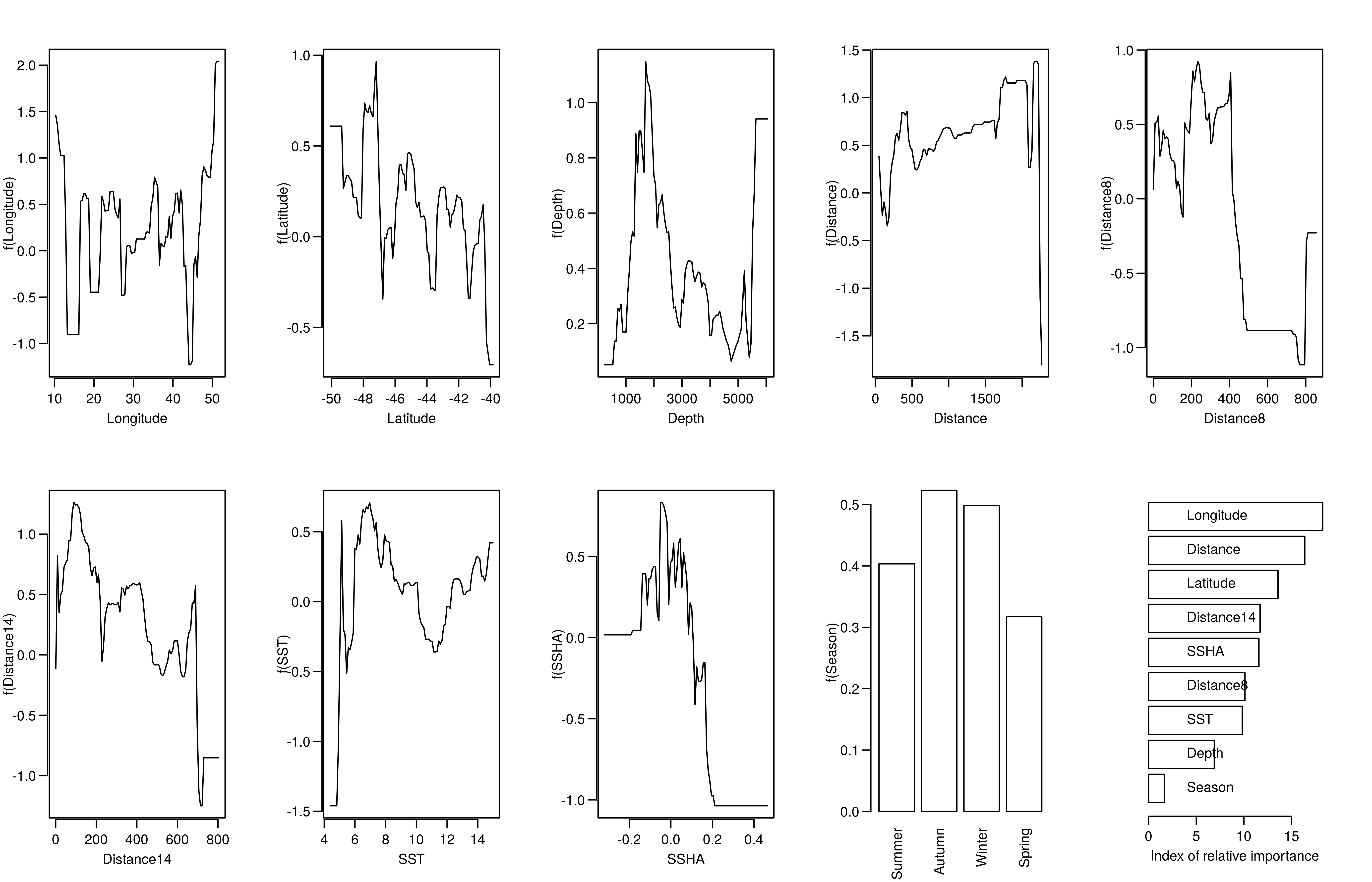

Supplement: S5 Fig — Distance represents distance from the study colony, Distance8 is the distance from the Subantarctic Front and Distance14 is the distance from the Subtropical Convergence Zone. The final panel shows the relative importance of all the predictors in terms of their influence on the predictive accuracy of the model. (TIF) [file pone.0152370.s005.tif]

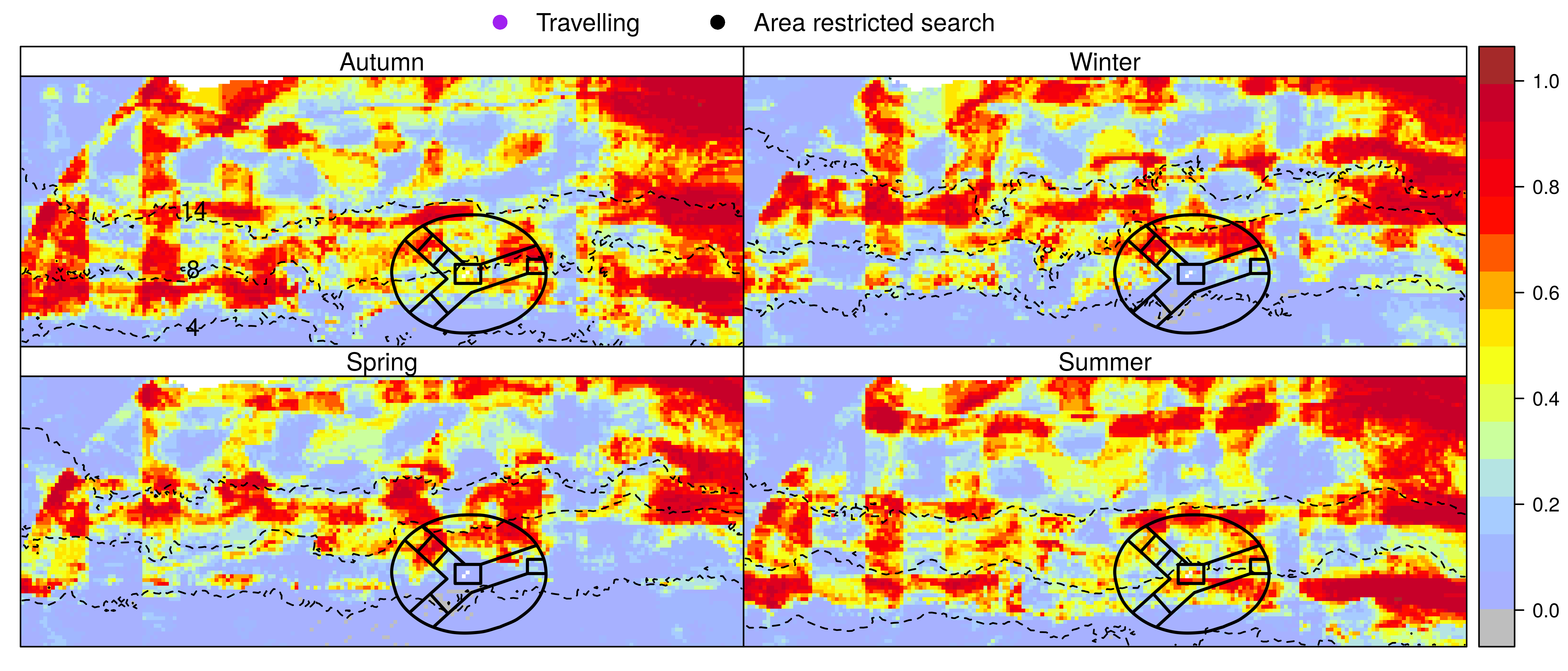

Supplement: S6 Fig — The dashed lines show the average surface locations of the Subtropical Convergence (STC), Subantarctic Front (SAF), and Antarctic Polar Front (APF), identified by the 14°C, 8°C, and 4°C sea surface temperature isotherms, respectively. (TIF) [file pone.0152370.s006.tif]
